# Skin Vascular Resistance Decreases during 5-HT-Induced Hypotension in the Rat

**DOI:** 10.3390/biomedicines11020547

**Published:** 2023-02-13

**Authors:** Benjamin Butler, Hannah Garver, Stephanie W. Watts, Gregory D. Fink

**Affiliations:** Department of Pharmacology and Toxicology, College of Osteopathic Medicine, Michigan State University, East Lansing, MI 48824, USA

**Keywords:** 5-HT, 5-HT_7_ receptor, skin circulation, skin temperature, hypotension

## Abstract

A recognized vasodilator, the infusion of 5-hydroxytryptamine (5-HT, serotonin) decreases blood pressure through the reduction of total peripheral resistance in the rat. It is not clear which vascular beds/tissues are responsible for this fall. We hypothesized that an increase in blood flow within the skin, measured as an elevated temperature (T) in the thermoregulatory tail and paws, enables at least part of 5-HT-induced reduction in blood pressure through active vasodilation. The temperature of thermoregulatory regions of the skin of an anesthetized male, Sprague Dawley rats were measured using a Optris PI640 thermal camera. The blood pressure of the animal and the temperature of each paw and four locations along the tail (TL1-4) were recorded before, during, and after the infusion of 5-HT at a rate of 25 mg/min into a femoral vein. Contrary to our hypothesis, the temperature of the paws and tail was stable before and during 5-HT infusion and actually increased during the 15-min recovery period. This finding suggests that hyperemia of the skin circulation is not necessary for the fall in blood pressure observed with infused 5-HT, but that a reduction in cutaneous vascular resistance plays a part in the fall in total peripheral resistance.

## 1. Introduction

Our laboratories have sought to understand how 5-hydroxytryptamine (5-HT, serotonin) reduces blood pressure. The potential clinical relevance of this finding, originally observed in man [1,2], has long been ignored even in the presence of the considerable literature supporting 5-HT-induced hypotension [3,4,5,6,7,8,9,10,11,12]. Understanding the mechanisms of this hypotension would not only allow for a potential insight into the causes of pathological hypotension, but it would also open doors for therapeutic intervention in hypertension, a field in which 5-HT pharmacology has not been well mined.

We have shown that chronic infusion of a low dose of 5-HT (25 μg/kg/min) reduces blood pressure in normal, freely moving rats [13] and nearly normalizes blood pressure of experimental and genetic models of rodent hypertension [14,15,16]. We have further shown that these effects are due to the activation of peripheral 5-HT_7_ receptors. Our pharmacological evidence for the 5-HT_7_ receptor being critical was strongly supported by findings that 5-HT infusion did not reduce blood pressure in 5-HT_7_ receptor KO rats [17]. Importantly, the vasculature is likely a site where 5-HT_7_ receptors cause hypotension since 5-HT infusion significantly reduces total peripheral resistance (TPR) [18] and 5-HT_7_ receptors have been identified in the vasculature [19].

However, we have not yet identified the most critical vascular beds responsible for the fall in TPR. Experiments using microspheres to quantify regional blood flow during acute and chronic 5-HT infusion generally show no change in the skin vasculature [4,20,21]. Isolated large and small arteries from a variety of vascular beds do not relax to 5-HT in vitro [18]. By contrast, veins do relax to 5-HT both in vitro [19] and in vivo [22]. However, venous dilation cannot account for the fall in TPR observed. Where, then, does the fall in TPR produced by 5-HT occur?

We recently demonstrated that 5-HT infusion causes a dramatic vasodilation in the rat hindquarters [23], and part of the hindquarters blood flow in the rats is directed to the skin (especially the tail). Experiments using microspheres to quantify regional blood flow during acute and chronic 5-HT infusion generally show no change in the skin vasculature [4,20,21]. In this study, we test the hypothesis that the non-hairy (glabrous) skin contributes to the 5-HT-induced fall in blood pressure through the dilation of cutaneous arteries. Experimentally, this would be observed as an increase in flow without an accompanying change in arterial pressure or a stable flow in the presence of a fall in arterial pressure during 5-HT infusion. It is clear that in both rats and humans, the cutaneous circulation can have a significant impact on total peripheral resistance. For example, the dramatic reduction in systemic vascular resistance that accompanies heat stress is predominantly caused by falls in cutaneous vascular resistance [24].

Heat loss estimated from skin temperature in thermoregulatory parts of the body is a widely used technique in physiology. An increase in skin temperature is a result of vasodilation, whereas a decrease in skin temperature signals vasoconstriction. Many investigators have demonstrated an association between 5-HT_7_ receptor activation and 5-HT-induced hypothermia based on pharmacological methods, and by knockout of the gene for this receptor (see [25] for review). However, it is not clear whether 5-HT-induced hypothermia results from vascular actions of the hormone or from other effects, such as activation of neuronal pathways involved in thermoregulation [25].

Previous Doppler flow experiments have been equivocal in their outcome and less than ideal because of the inability to measure several regions of interest (paws, tail) at the same time. We turned to thermography as an alternative approach. Since vasodilation in the skin of the tail and paws is one of the primary means by which rats shed heat, as noted above, skin temperature (T) is an excellent surrogate for blood flow [26,27]. This technique also allows for the monitoring of multiple cutaneous regions at one time [28,29]. Finally, thermography allows for the monitoring of T longitudinally throughout an experiment. Thermography was combined with the continuous measurement of blood pressure such that the temporal relationship between cutaneous T and blood pressure could be well-defined.

## 2. Results

**Figure 1:**
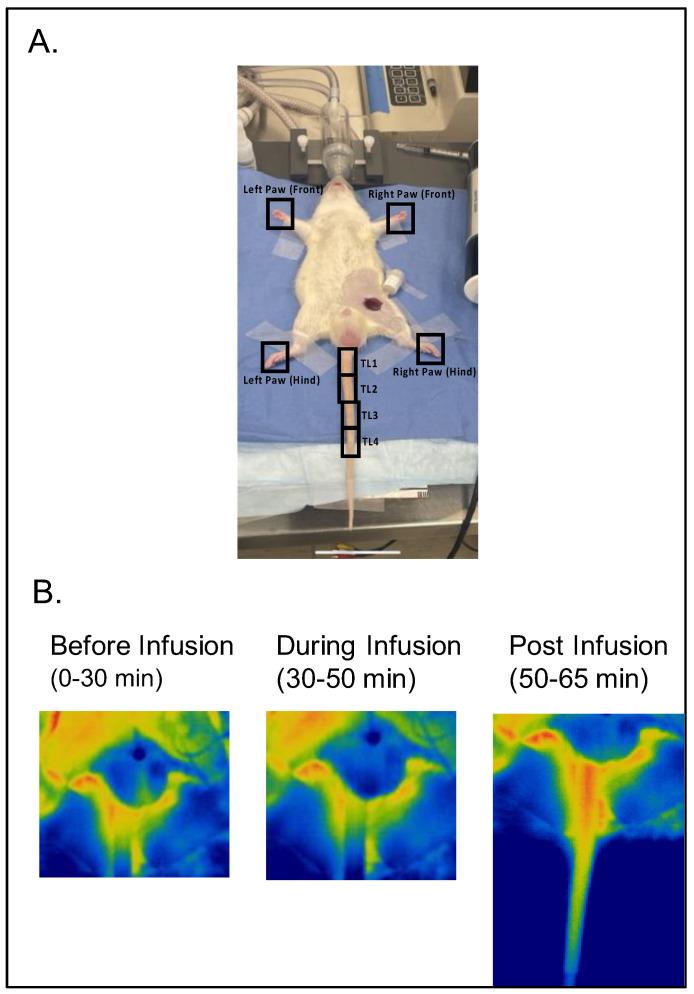
(**A**) Illustration of experimental setup with regions of interest (four paws and four tail segments as TL1, TL2, TL3, and TL4) marked with a black square/rectangle. (**B**) Representative images of the tail of the same rat before, during, and after 5-HT infusion (timing in protocol). Warm colors indicate greater flow.

Figure 1A illustrates the experimental setup with regions of interest—the paws and tail segments divided into four segments: TL1, TL2, TL3, and TL4—defined for analyses. Figure 1B depicts images of the rat tail (base primarily) before, during, and after infusion of 5-HT.

**Figure 2:**
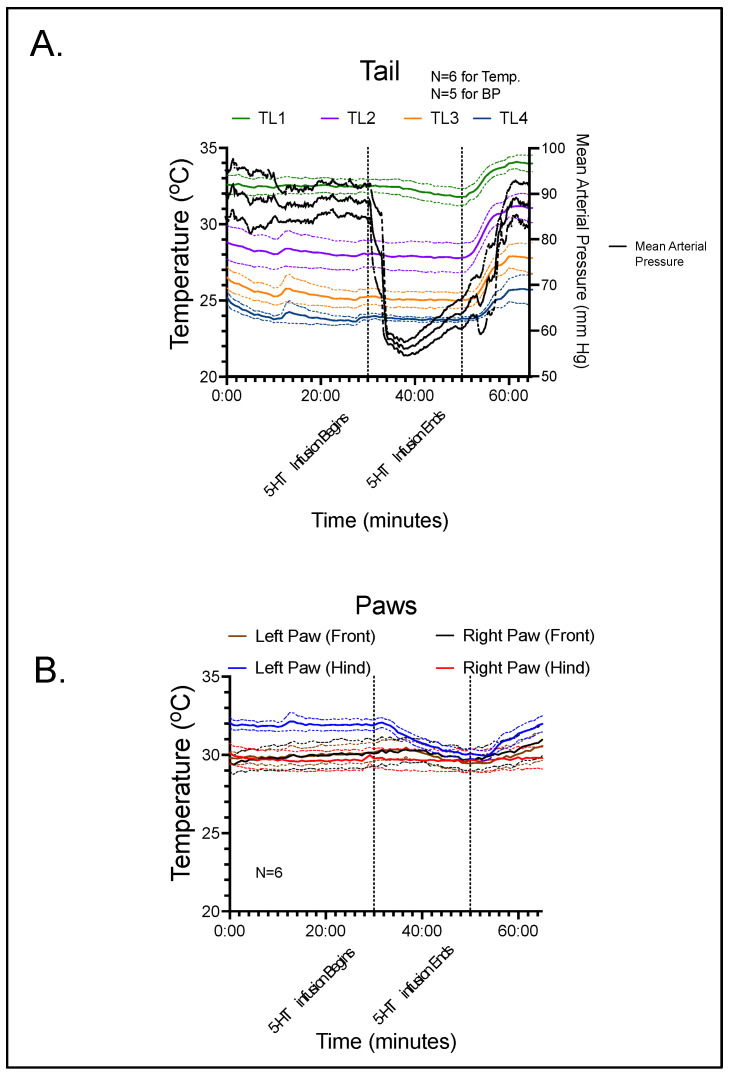
(**A**) Blood pressure and thermographic data for segments of the rat tail during the course of the experiment. (**B**) Thermographic data for the four paws of the rat during the course of the experiment. Lines represent means with shaded area the standard deviation for the number of rats reported.

Figure 2 combines thermography with continuous blood pressure data for the tail (Figure 2A) and thermography only for the paws (Figure 2B). The black line in Figure 2A illustrates the mean arterial pressure (MAP; mm Hg) of five (5) rats receiving 5-HT infusion. In one rat (6th), a stable recording could not be obtained, and thus the blood pressure of this rat is not reported. Mean arterial blood pressure was measured throughout the duration of the experiment to allow the animal to serve as its own control. A reading was taken every 10 s before, during, and after infusion of 5-HT. The baseline average for the five rats that were measured was 88.4 ± 3.6 mmHg, which remained largely consistent until 5-HT infusion was initiated. 5-HT infusion, begun at 30 min, caused a profound hypotension. The nadir was 56.3 ± 1.5 mmHg (*p* < 0.001 compared to control), meaning that 5-HT infusion caused over a 30 mmHg peak fall in blood pressure. This is consistent with published work and confirms that 5-HT at this infusion rate/dosing causes hypotension. At the end of the 20-min infusion period, mean arterial pressure was 63.6 ± 3.1 mmHg (*p* < 0.001 compared to control), which rose to 88.1 ± 3.7 mmHg (not significantly different from control) following the 15-min recovery time.

Temperature data were measured continuously, with a measurement being recorded every 50 s. For the paws of the animal, the mean baseline was recorded as 32.0 °C for the left rear, 30.2 °C for the left front, 29.8 °C for the right rear, and 30.1 °C for the right front. Baseline values for the tail (TL1-4) were 32.5, 28.1, 25.3, and 24.0 °C, respectively. During the peak hypotensive effect of 5-HT, the temperature of the left rear, left front, right rear, and right front paws changed only slightly: −1.7 ± 0.58, 0.05 ± 0.21, −0.13 ± 0.12, and 0.15 ± 0.21 °C, respectively. For the four locations along the tail, the values at peak hypotensive effect consistently fell: −0.20 ± 0.23, −0.16 ± 0.20, −0.20 ± 0.19, and −0.17 ± 0.08 °C, respectively. At the end of the 5-HT infusion period, T continued to decline slightly at all sites (see Figure 2). However, none of these changes were statistically significant. During the recovery period, the temperature of the four paws increased to above pre-infusion values: 0.03 ± 0.55 for the left rear, 0.33 ± 0.36 for the left front, 0.00 ± 0.13 for the right rear, and 0.85 ± 0.40 °C for the right front, respectively. Along the four tail locations, the temperature differences between recovery and pre-infusion values were 1.48 ± 0.35, 2.97 ± 0.58 (*p* < 0.05), 2.50 ± 0.44 (*p* < 0.05), and 1.75 ± 1.00 °C, respectively.

During 5-HT infusion, temperature of the tail did not increase significantly in the paws or any segment of the tail when compared to baseline. This suggests that an increase in blood flow in the thermoregulatory cutaneous vasculature does not occur during 5-HT-induced hypotension in the rat. However, the stable cutaneous blood flow during the rapid and large decrease in blood pressure caused by 5-HT indicates that vascular resistance in the cutaneous bed was dramatically decreased. Interestingly, cessation of 5-HT infusion was associated with a statistically significant increase in temperature in all tail segments (TL1, TL2, TL3 and TL4) and a modest increase in the paws as well.

## 3. Discussion

This study tested the hypothesis that 5-HT increases skin blood flow through active vasodilation, and that this vasodilation contributes to the fall in blood pressure observed during 5-HT infusion. Our findings are consistent with 5-HT causing cutaneous vasodilation but do not prove that such vasodilation is necessary for 5-HT induced hypotension.

At least as measurable by thermography, 5-HT infusion was associated with a marked fall in blood pressure without any change in blood flow in the tail or paws. This result implies either active cutaneous vasodilation by 5-HT, or vascular autoregulation (a fall in resistance in response to decrements in perfusing pressure) in response to the decline in blood pressure. Alone, our results do not allow us to distinguish between these possibilities. However, the cutaneous vascular bed is known to exhibit very poor autoregulatory capacity [30]. Thus, we conclude that the decrease in cutaneous vascular resistance during 5-HT infusion is caused by 5-HT acting directly on 5-HT_7_ receptors on cutaneous arteries and arterioles. This is predicated on the relationship that pressure (P) is equal to flow (F) times resistance (R) (P = F × R). If F—using T as a surrogate—does not change during an intervention, then pressure and resistance must change in the same direction and magnitude. Although we propose that the fall in cutaneous resistance during 5-HT infusion is due to the direct effects of 5-HT on 5-HT_7_ receptors on cutaneous arteries, we cannot rule out the possibility that 5-HT infusion induces the release of another endogenous cutaneous vasodilator; this idea will require further investigation. Furthermore, since the activation of 5-HT_1A_, 5-HT_3_, 5-HT_7,_ and 5-HT_2_ receptors can all affect thermoregulation, additional studies with specific antagonists are necessary to establish the relative role of each in our 5-HT infusion model.

The tail vasculature is under significant centrally derived sympathetic control and has the potential to conduct up to 10% of cardiac output when dissipation of heat is necessary [31]. But the hypotension associated with 5-HT infusion would be expected to produce a baroreflex-induced increase in cutaneous vascular resistance, not the decrease we observed. The rapid waning of this baroreflex-mediated sympatho-excitation and associated cutaneous vasoconstriction likely explains the mild “overshoot” in tail temperature (and thus blood flow) after terminating 5-HT infusion. This possibility could be investigated in future studies by repeating the current protocol in rats in which sympathetic innervation to the tail was removed prior to 5-HT infusion.

We acknowledge a few limitations of the present study. First, our studies were performed under isoflurane anesthesia. Therefore, the well-known suppression of central thermoregulation during isoflurane anesthesia could have affected our findings. However, instead of investigating thermoregulation per se, we were using ear and tail temperatures merely as a surrogate for skin blood flow, specifically for skin blood flow changes due to direct vascular actions of 5-HT. Thus, isoflurane anesthesia may actually be an advantage for our purposes since it would likely minimize any potential central actions of infused 5-HT on skin blood flow. Second, we studied only males. Males and females show a similar fall in BP to infused 5-HT as well as a dependence on the 5-HT_7_ receptor for this fall [17]. As such, we considered the animal use principle of not using more animals than needed to come to a scientific conclusion and used only males. Nevertheless, investigating the impact of 5-HT on cutaneous blood flow in females needs to be done. Another limitation is that our conclusions only can be applied to *acute* 5-HT infusion. Our studies have largely been dedicated to understanding the effects of 5-HT infusion on blood pressure over longer periods, i.e., from a week to 30 days. Hypotension during these chronic infusions is similarly dependent on the 5-HT_7_ receptor as is acute 5-HT-infused hypotension: both are abolished by pharmacological antagonism [22] or genetic removal [17] of the 5-HT_7_ receptor. Similarly, the 5-HT_1A/7_ agonist 5-carboxyadmidotryptamine causes a 5-HT_7_ receptor dependent venous relaxation and hypotension [19]. However, long-term agonist infusions will typically engage both complementary and opposing physiological responses that complicate efforts to establish precise causes of the response under investigation.

## 4. Materials and Methods

### 4.1. Animals

Male Sprague Dawley rats from Charles River Laboratories (Mattawan, MI, USA) of 240–270 g were used. This work was reviewed and approved by the Michigan State University Institutional Animal Care and Use Committee through protocol PROTO201800191.

### 4.2. Anesthesia

Animals were initially induced with 5% Isoflurane and O_2,_ and the weight was recorded. The abdominal area above where the femoral artery is located was shaved. Isoflurane was reduced to 2%, and the rat was transferred to a solid surgical table with a 42 °C water-based heating pad underneath a surgical cloth. The rat was secured using biocompatible tape in a spread-eagle position with the tail placed directly away from the body. The ambient temperature in the laboratory was 22 ± 1.0 °C.

### 4.3. Surgery

A telemeter probe catheter (Data Sciences International, Minneapolis, MN, USA) was implanted into a femoral artery (the transmitter was left outside the rat’s body) to measure blood pressure continuously through the experiment. The mean arterial pressure was recorded on DSI Data Art 4.31. Thermography data were recorded through PIX Connect Software on an HP Spectre Laptop (i7 Intel).

### 4.4. Imaging System

An Optris PI640 camera was connected to a 2021 Spectre laptop and the PIX Connect Software was used to capture images. The camera was mounted 30 inches above the animal using a ring stand apparatus. The Optris PI640i infrared imager camera has a 640 × 480 resolution, 40 mK NETD, USB 2.0 interface, process interface, environmental protection IP 67, and an automatic internal calibration system. At ambient temperatures within the range of 18–28 °C, the system accuracy of the camera is ±2 °C.

### 4.5. Data Acquisition

Eight (8) regions of interest were imaged: all four paws and four tail regions [tail location, TL: TL1, TL2, TL3, and TL4]. This was done by placing a region of interest box within the PIX Connect Software over the region of interest. The same size boxes were used in the six (6) different experiments reported in this manuscript. Data were continually acquired through three different phases of the experiment: prior to 5-HT infusion, during 5-HT infusion, and after 5-HT infusion. In this manner, the animal served as its own control.

### 4.6. Baseline, 5-HT Infusion, and Post-Infusion

Once the rat was secured and the catheter implanted, a 30-min period of baseline measurements was obtained. At 30 min, an infusion of 5-HT at a rate of 25 mg/min was carried out by means of an infusion pump (KD Scientific 780200). This delivered the 25 mg/kg/min dose reported previously to cause hypotension in the rat [13]. With this same dose, the 5-HT infusion was continued for 20 min. At the end of 20 min, the infusion pump was stopped, and a 15-min period of post-infusion data was collected. Euthanasia was performed by cardiac exsanguination while the subjects were still under anesthesia.

### 4.7. Data Analyses and Statistics

Absolute magnitudes of mean arterial blood pressure and segmental temperatures were transferred into Graph Pad Prism 9.0 (La Jolla, CA, USA) as individual values for each rat. The graphs presented are the means surrounded by the shaded standard error of the mean. Differences in mean arterial pressure and T during the pre-infusion, peak blood pressure response, end of infusion, and end of recovery periods were analyzed using a one-way repeated measure ANOVA, followed by Dunnett’s test for pre-defined multiple comparisons. A *p*-value < 0.05 was considered statistically significant.

## Data Availability

Raw data for this study are available upon reasonable request.
